# The Accumulation of VEGFA in the Glomerular Basement Membrane and Its Relationship with Podocyte Injury and Proteinuria in Alport Syndrome

**DOI:** 10.1371/journal.pone.0135648

**Published:** 2015-08-14

**Authors:** Haiyan Wang, Zhihui Yue, Jinlang Wu, Ting Liu, Ying Mo, Xiaoyun Jiang, Liangzhong Sun

**Affiliations:** 1 Children’s Kidney Disease Center, Department of Pediatrics, The First Affiliated Hospital, Sun Yat-sen University, Guangzhou, P. R. China; 2 Department of Pediatrics, Sun Yat-sen Memorial Hospital, Sun Yat-sen University, Guangzhou, P. R. China; 3 Department of Electron Microscopy, Zhongshan School of Medicine, Sun Yat-sen University, Guangzhou, P. R. China; University of Utah School of Medicine, UNITED STATES

## Abstract

The pathogenesis of proteinuria in Alport syndrome (AS) remains unclear. Vascular endothelial growth factor A (VEGFA) is a key regulator of the glomerular filtration barrier (GFB). This study explored the expression of VEGFA in the glomeruli and its accumulation in the glomerular basement membrane (GBM) and their relationship with podocyte injury and proteinuria in Alport syndrome (AS). Clinical data and renal tissues of control patients (11 cases) and AS patients (25 cases) were included. AS patients were further divided into 2 groups according to the quantities of their urinary protein: mild to moderate proteinuria group (proteinuria <50 mg/kg/d, 15 cases) and heavy proteinuria group (proteinuria ≥50 mg/kg/d, 10 cases). The expression and distribution of VEGFA and VEGF receptor 2 (VEGFR2) in the GFB, the phosphorylation of VEGFR2 (p-VEGFR2) and nephrin (p-nephrin), and the expression of synaptopodin and nephrin in the glomeruli were detected by immune electron microscopy and/or immunofluorescence, and their relationships to proteinuria in AS patients were analyzed. The accumulation of VEGFA in the GBM was increased in AS patients. The expression of VEGFA and the levels of p-VEGFR2 and p-nephrin in glomeruli were increased and were positively correlated with the degree of proteinuria in AS patients. The expression of synaptopodin and nephrin were decreased and were negatively correlated with the degree of proteinuria in AS patients. The over expressed VEGFA in the glomeruli and its accumulation in the GBM may activate the VEGFA-VEGFR2 and nephrin signaling pathways and lead to podocyte injury and occurrence of proteinuria in AS.

## Introduction

Alport syndrome (AS) is the most common inherited progressive glomerulonephritis in children and it is caused by defects in type IV collagen (COL IV α3, α4 or α5 chain) in the glomerular basement membrane (GBM) [1, 2]. The presence of proteinuria is an important risk factor of disease progression [3]. However, the pathogenesis of proteinuria in AS remains unclear. Dysfunction of the glomerular filtration barrier (GFB) is presumably associated with the development of proteinuria. Vascular endothelial growth factor A (VEGFA) plays an important role in the maintenance of GFB function. Both the over-expression and the down-regulation of VEGFA could cause GFB injuries and the occurrence of proteinuria [4, 5]. Vascular endothelial growth factor receptor 2 (VEGFR2) is the main receptor for biological mediation function of VEGFA [6]. It has been reported that VEGFR2 could bind to the intracellular domain of nephrin [7]. The phosphorylation of Tyr1214 within VEGFR-2 triggers the recruitment of Nck and the activation of Fyn, which can mediate the tyrosine phosphorylation of nephrin and initiates signaling events that might dynamically integrate podocyte actin cytoskeleton dynamics and podocyte intercellular junction formation [8, 9]. The present study explored the pathogenesis of proteinuria in AS by detecting the expression of VEGFA and VEGFR2 in glomeruli, the accumulation of VEGFA in GBM, the phosphorylation of VEGFR2 and nephrin, and the association of all of these markers with podocyte injury and the development of proteinuria in AS patients.

## Materials and Methods

### Subjects

AS patients who were diagnosed in our hospital between October, 2010 and March, 2014 were included in this study. Diagnosis of AS was conducted in accordance with the criteria reported by Flinter [10], AS can be diagnosed when one of the following conditions is met: (1) abnormal staining of the COL IV α3 or α5-chain in GBM or in skin basement membrane, (2) typical ultrastructural changes visible with an electron microscope, and (3) mutations in COL4 A3-5 genes. The control patients (group 1) were those who underwent nephrectomy, and primary and secondary glomerular nephropathy were excluded. AS patients were further divided into 2 groups according to their degree of proteinuria: group 2, patients with mild and moderate proteinuria (urinary protein <50 mg/kg/d) and group 3, patients with heavy proteinuria (urinary proteinuria ≥50 mg/kg/d).[11] The clinical and pathological data and renal tissues were collected from AS patients and control patients.

The study was conducted in accordance with the principles outlined in the Declaration of Helsinki with approval from the ethics committee of the First Affiliated Hospital, Sun Yat-Sen University. Written informed consent was obtained from the subjects or their parents.

### Antibodies and Reagents

Antibodies were purchased from the following sources: VEGFA, synaptopodin and p-VEGFR2 (Y-1214) (Santa Cruz, USA); nephrin, VEGFR2 and p-nephrin (Y-1217) (Abcam, USA); COL IV α1, α3 antibodies and α5 antibodies (Wieslab Alport’s syndrome kit, Sweden); FITC-conjugated goat anti-rabbit antibody (Sigma Aldrich, USA), FITC-conjugated pig anti-mouse antibody (Dako, Denmark); nanogold-conjugated goat anti-rabbit antibody (gold immunoprobe is 1.4 nm in diameter) and HQ silver enhancement kit (Nanoprobes, NY, USA); and goat serum (Boster, China).

### Experimental Protocols

The expression levels of synaptopodin, nephrin, VEGFA and VEGFR2 and the levels of p-VEGFR2 and p-nephrin within the glomeruli were detected by immunofluorescence. The expression and distribution of VEGFA and VEGFR2 were detected by immune electron microscopy. Vehicle control samples were set by adding tris buffered saline (TBS) without either primary or secondary antibodies during the detections. The protocols were as follows.

Immunofluorescence: Fresh renal tissues were immersed in 50 μM pervanadate for 15–20 mins and were frozen sectioned (4 μm) at -20°C. The sections were incubated in blocking goat serum [5% (v/v) goat serum, TBS (150 mM NaCl, 20 mM Tris)] at room temperature for 30 mins and then incubated with the following primary antibodies at 4°C overnight: anti-synaptopodin (1:100), anti-nephrin (1:100), anti-VEGFA (1:50), anti-VEGFR2 (1:100), anti-p-VEGFR2 (Y-1214) (1:50), anti-p-nephrin (Y-1217) (1:100). COL IV α1, α3 and α5 antibodies (1:20, 1:20 and 1:50, respectively) were incubated with the sections at 37°C for 1–2 hours. Sections were then rinsed with TBS (5 mins × 3 times) and incubated with FITC-conjugated anti-rabbit antibodies (1:100) or FITC-conjugated anti-mouse antibodies (1:20) at 37°C for an hour; they were rinsed with TBS (5 mins × 3 times) before observation and photographed with an immunofluorescent microscope (Olympus BX51WI, Japan). Quantitative analyses of immunofluorescence images were performed on Image pro-plus 6 software. Three to five tangent glomeruli were randomly selected in each specimen. Optical density (OD) of each glomerulus was normalized by glomerular area, and then, the mean OD of these glomeruli was taken to statistical analysis.

Immune electron microscopy: Fresh renal tissues were pre-fixed [2% paraformaldehyde, 15% picric acid (v/v), 0.03% glutaraldehyde (v/v), and sodium phosphate buffer (NaPB, 20 mM NaH_2_PO_4_ and 10 mM Na_2_HPO_4_)] for 3–4 hours and were rinsed with NaPB 3 times. Then, the samples were put into 10% and 20% sucrose buffer [10% and 20% sucrose (w/v) in NaPB] and cryoprotectant buffer [30% sucrose (w/v) and 25% glycerol (v/v) in NaPB] for 10, 10 and 30 mins subsequently. Then, the samples were placed in liquid nitrogen within cryoprotectant buffer. The samples were cut in 50 μm thick slices by cryosection and were blocked with 20% (v/v) goat serum for 30 mins. They were then incubated with primary antibodies [anti-VEGFA (1:50) and anti-VEGFR2 (1:100)] at 4°C with shaking for 24 hours. After 3 rinses with TBS, the samples were incubated with nanogold-conjugated goat anti-rabbit antibody (1:100) at 4°C with overnight shaking. Then, the samples were rinsed with NaPB 3 times and postfixed with 1% glutaraldehyde for 10 mins; they were HQ silver enhanced for 8–15 mins. The remaining processes were performed as previously described [12]. Then, the samples were observed under a transmission electron microscope (Tecnai G2 Sptrit BioTWIN, FEI, OR, USA).

### Statistical Analysis

Statistical analyses were performed with SPSS 13.0 software. The indexes of age and immunofluorescence in each group were expressed as the median (maximum, minimum). The indexes of data with normal distribution were expressed as mean ± standard deviation (SD). Data were analyzed using the Kruskal-Wallis test within 3 groups, and the Bonferroni test was used in between-group comparison (statistical significance was considered to be *p*<0.05/3 = 0.017). The Spearman test was used for the correlation analysis, and statistical significance was considered to be *p*<0.05.

## Results

### Clinical data

Twenty five AS patients (19 male, 4 female) were included in the present study (Table 1). Fifteen patients (12 male, 3 female) had mild to moderate proteinuria (group 2, urinary protein was 18.45±6.04 mg/kg/d) and their median age at renal biopsy was 3.5 years (1.9 years, 11.0 years). Ten patients (9 male, 1 female) had heavy proteinuria (group 3, urinary protein was 108.45±45.43 mg/kg/d) and their median age at renal biopsy was 10.5 years (5.9 years, 17.0 years). Hearing impairment was observed in 5 of the 25 AS patients, one in group 2 and four in group 3. Optical injury was not observed in any patient in both groups.

**Table 1:** The clinical and pathological features of the 25 AS patients.

Patients	Sex	Age at diagnosis (year)	Quantity of proteinuria (mg/kg/d)	Scr (μmol/l)	Bun (mmol/l)	GFR (ml/min/1.73m^2^)	Extra-renal involvement (ears/eyes)	Renal COL4 α3 GBM/TBM	Renal COL4 α5 GBM/BC/TBM	Renal pathology changes observed by LM	Renal pathology changes observed by TEM
1	F	2.7	16.4	24	5.0	104.0	Yes/No	± / ±	± / ± / ±	MsPGN (mild)	Thinning in part of GBM
2	F	3.5	14.7	29	4.7	190.8	No/No	+ / ±	± / ± / -	MCD	Thinning in part of GBM
3	M	1.9	17.1	20	2.6	137.0	No/No	- / -	- / - / -	MCD	Uneven density, irregular thickening and thinning in GBM
4	M	3.3	21.8	20	4.0	182.4	No/No	+ / -	- / - / -	MsPGN (mild)	Thinning in part of GBM
5	M	3.9	35.9	29	4.1	151.1	No/No	- / -	- / - / -	MsPGN (mild)	Uneven density, irregular thickening and thinning in GBM
6	M	2.0	21.4	26	6.1	240.0	No/No	- / -	- / - / -	MsPGN (mild)	Uneven density, irregular thickening and segmental layering in GBM
7	M	8.8	15.2	33	4.8	133.9	No/No	± / ±	- / - / -	MsPGN (mild)	Segmental thickening, thinning and layering in GBM
8	F	10.8	22.7	34	4.6	79.2	No/No	+ / +	± / ± / +	FSGS	Proliferation in mesangial, and thickness in GBM
9	M	4.5	15.7	34	5.6	91.2	No/No	- / -	- / - / -	MCD	Uneven density, irregular thickening and widely layering in GBM
10	M	2.3	13.3	23	3.3	117.2	No/No	± / -	- / ± / -	MCD	No glomerli was observed
11	M	3.0	12.2	25	6.2	132.4	No/No	- / -	- / - / -	MsPGN (mild)	Uneven density, irregular thickening and partial thinning in GBM
12	M	3.5	21.8	23	2.9	121.2	No/No	- / -	- / - / -	MCD	Uneven density, irregular thickening and occasional thinning in GBM
13	M	4.1	21.2	27	3.7	146.4	No/No	± / -	- /±/ -	MsPGN (mild)	Partial thinning in GBM, MCD
14	M	11.0	14.7	47	4.2	113.4	No/No	- / -	- / - / -	MsPGN (mild)	Uneven density, irregular thickening and occasional thinning in GBM
15	M	4.8	12.8	44	6.2	89.7	No/No	± /±	± / ± / -	MCD	Widely thinning in GBM, MCD
16	F	8.2	67.3	128	8.2	52.8	No/No	- / -	- /±/ -	FSGS	Thinning, tearing and mesangial insertion in part of GBM
17	M	5.9	58.4	27	4.5	199	No/No	- / -	- / - / -	MsPGN (mild)	Tearing in segmental GBM
18	M	12.1	81.9	72	11.5	97.2	Yes/No	- / -	- / - / -	MsPGN (mild)	Uneven density and irregular thickening in part of GBM
19	M	12.9	173.5	90	17.7	32.6	Yes/No	- / -	- / - / -	FSGS	Widely thickening, tearing and layering in GBM
20	M	9.7	67.7	57	14.1	80.6	No/No	- / -	- / - / -	MsPGN (mild)	Widely thickening, tearing and layering in GBM
21	M	17	164.8	74	6.7	86.9	Yes/No	- / -	- / - / -	MsPGN (mild)	Widely thickening, tearing and layering in GBM
22	M	9.3	72.4	30	10.4	210	No/No	- / -	- / - / -	FSGS	Widely thickening, tearing and layering in GBM
23	M	11.3	124.9	95	7.8	81.1	Yes/No	- / -	- / - / -	MsPGN (mild)	Widely thickening, tearing and layering in GBM
24	M	12.3	110.0	46	5.6	175.5	No/No	- / ±	- / - / -	MsPGN (mild)	No glomerli was observed
25	M	6.0	163.6	39	8.3	81.4	No/No	+ / +	+ / + / +	MsPGN (mild)	Uneven density, irregular thickening and tearing in part of GBM

Note: ALB, albumin; BC, Bowman's capsule; BUN, blood urine nitrogen; COL4, type IV collagen; FSGS, focal and segmental glomerular sclerosis; GBM, glomerular basement membrane; GFR, glomerular filtration rate; GS, global sclerosis; LM, light microscopy; MCD, minimal change of disease; MsPGN, mesangial proliferation glomerulonephritis; TBM, tubular basement membrane; TEM, transmission electron microscopy; -, negative; ±, non consecutive positive; +, positive.

The levels of Scr (65.8±32.3 μmol/l) and blood urine nitrogen (BUN, 9.5±4.05 mmol/l) of patients in group 3 were higher than those (Scr 29.6±8.03 μmol/l and BUN 4.5±1.15 mmol/l) of patients in group 2 (*p* values were <0.001 both, Table 2). Both Scr and BUN were positively correlated with the levels of proteinuria (both of *p* values were 0.001). The mean glomerular filtration rate (GFR, which was calculated by the Schwartz formula) was 135.3±42.93 ml/min/1.73 m^2^ in group 2 and 109.9±62.08 in group 3. The differences in GFR between two groups and the relationships between GFR and proteinuria were both of no significance (Table 2).

**Table 2:** Comparison of the indexes of renal impairments between two groups of AS patients.

	Median age (min, max) (year)	Mean proteinuria ± SD (mg/kg/d)	Mean Scr ± SD (μmol/l)	Mean BUN ± SD (mmol/l)	Mean GFR ± SD (ml/min/1.73m^2^)
Group 2	3.5 (1.9, 11.0)	18.45±6.04	29.6±8.03	4.5±1.15	135.3±42.93
Group 3	10.5 (5.9, 17.0)	108.45±45.43	65.8±32.3	9.5±4.05	109.9±62.08
*p*	< 0.001	< 0.001	< 0.001	< 0.001	0.234

Note: group 2, AS patients with mild and moderate proteinuria; group 3, AS patients with heavy proteinuria; Scr, serum creatinine; BUN, blood urine nitrogen; GFR, glomerular filtration rate.

Renal pathology under light microscopy were mild mesangial proliferative glomerulonephritis (MsPGN, 15/25 patients) and minimal change disease (MCD, 6/25 patients) and focal segmental glomerulosclerosis (FSGS, 4/25 patients). Extensive or segmental irregular thickening, attenuation, tearing and layering in GBM were observed under electron microscopy in 17 available patients. Complete loss of immunofluorecent staining of type IV collagen (COL4) α5 chain in GBM was observed in 20/25 patients (Table 1).

Eleven patients were included in the control group (2 male and 9 female: Wilm’s tumor 5 cases, renal carcinoma 3 cases, duplex kidney 2 cases and pyelo carcinoma 1 case); the median age at nephrectomy was 6.7 years (0.2 years, 42 years). There was no significant age difference between control patients and AS patients. Two of the AS specimens with heavy proteinuria exhibited focal segmental glomerulosclerosis, the remaining specimens showed minimal change disease or mild-to-moderate mesangial proliferative glomerulonephritis.

### Experimental Data

#### The expression and distribution of VEGFA and VEGFR2, the phosphorylation of VEGFR2 in glomeruli, and their association with proteinuria, Scr, BUN and GFR in AS patients

**Fig 1:**
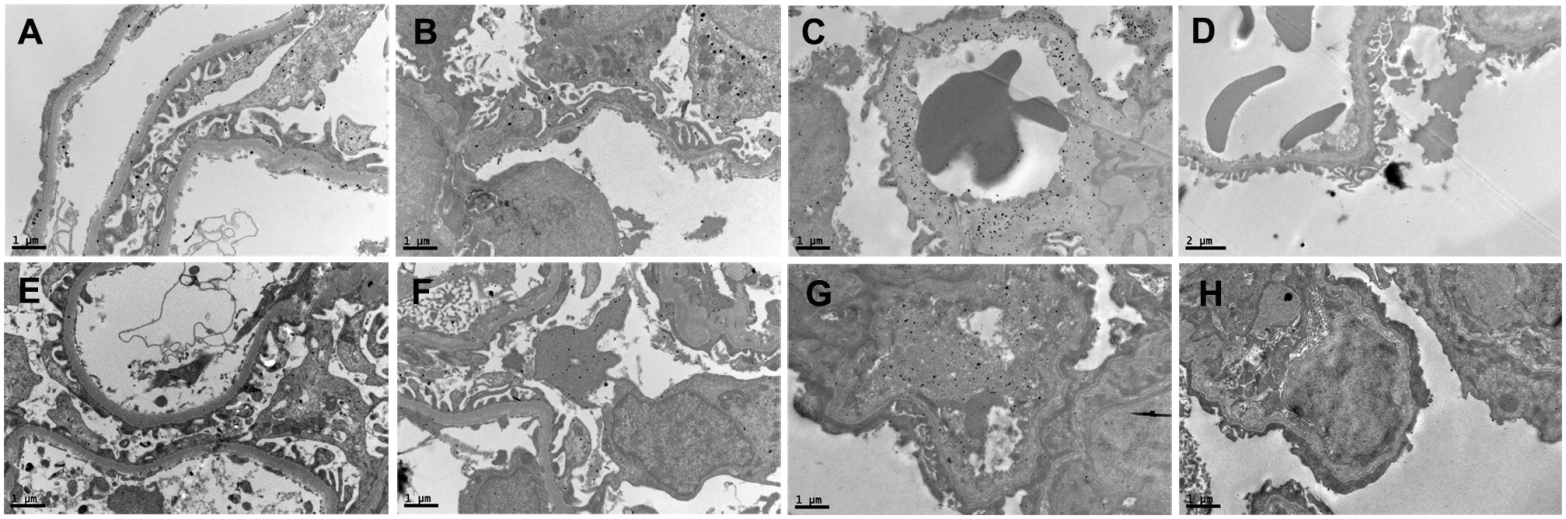
The expression and distribution of VEGFA (a, b and c) and VEGFR2 (e, f and g) in glomeruli (immune electron microscope, a and e: control patients; b and f: AS patients with mild proteinuria. c, d, g and h: samples were from the same AS patient with heavy proteinuria. d and h: vehicle controls by adding primary antibodies of VEGFA and VEGFR2 repectively but not secondary antibody). The expression of VEGFA was observed in three layers of the GFB (podoyctes, GBM and glomerular endothelial cells); patients with heavy proteinuria showed more VEGFA accumulation in the GFB (a, b and c). The expression of VEGFR2 was detected both in the podocytes and endothelial cells, and the expression of VEGFR2 increased in AS patients with heavy proteinuria (e, f and g). Black particle was not observed in vehicle controls (d and h) (black particles indicated VAGFA or VEGFR2. Magnifications: a-h were 9700, bar: 1 μm).

Immune electron microscopy revealed that VEGFA was detected in the three layers of the GFB (podocytes, GBM and endothelial cells); the accumulation of VEGFA within the GBM was increased in AS patients and was apparent in patients with heavy proteinuria (Fig 1a–1c). VEGFR2 expression was observed both in endothelial cells and in podocytes (Fig 1e–1g).

**Fig 2:**
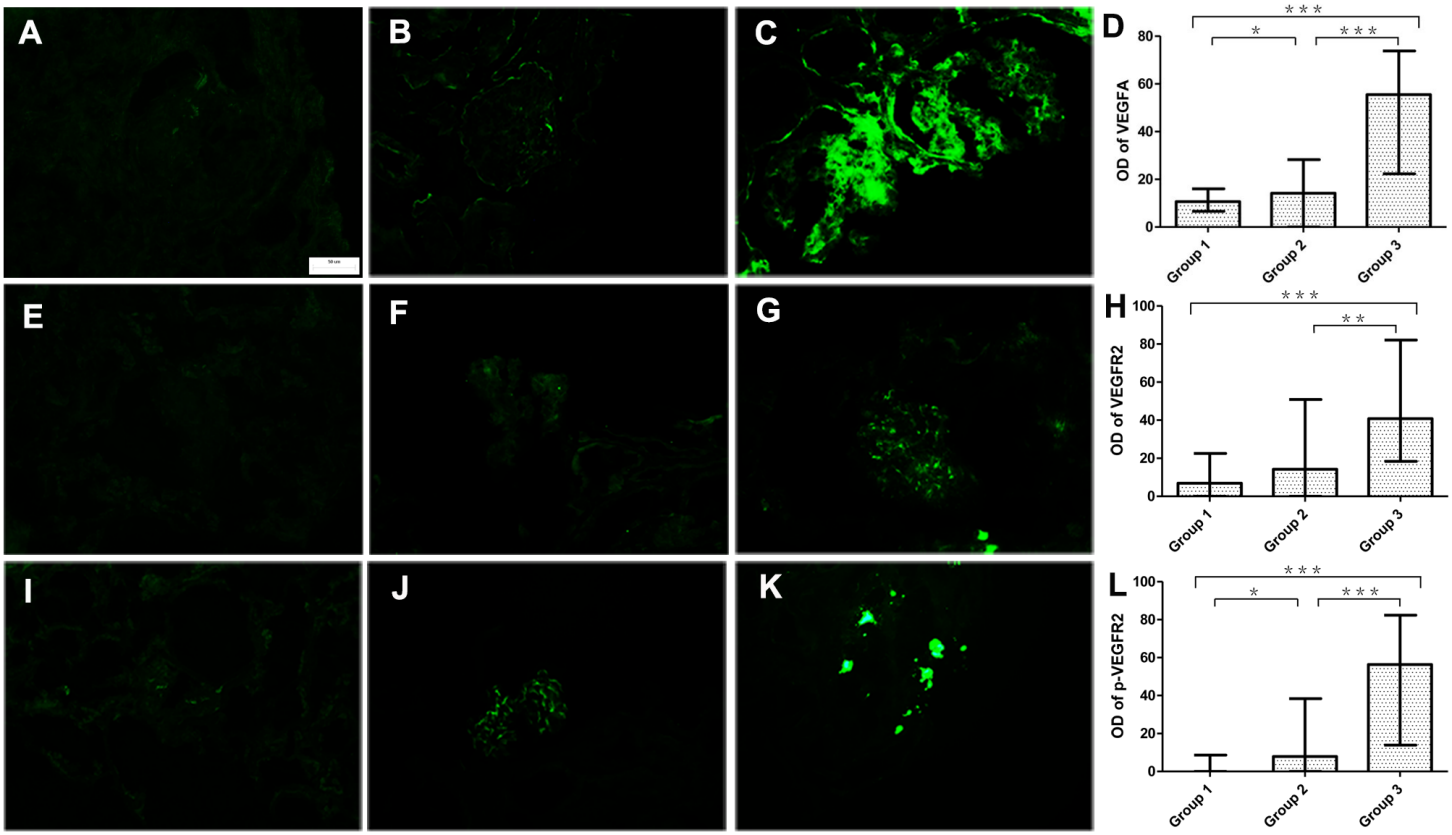
The expression of VEGFA, VEGFR2 and p-VEGFR2 in glomeruli (immunofluorescence, a, e and i: control patients; b, f and j: AS patients with mild proteinuria; and c, g and k: AS patients with heavy proteinuria). The expression of VEGFA in glomeruli was faint or undetectable in control patients (A), slightly increased in AS patients with mild proteinuria (b), and prominently up-regulated in AS patients with heavy proteinuria (c). The expression of VEGFR-2 was faint in control patients (e), slightly up-regulated in AS patients with mild proteinuria (f) and prominently increased in AS patients with heavy proteinuria (g). The level of p-VEGFR2 could not be detected in the control group (i), was faint in AS patients with mild proteinuria (j), and was apparently increased in AS patients with heavy proteinuria (k). d, h and l were the comparison analyses of the immunofluorescence optical density of VEGFA, VEGFR2 and p-VEGFR2 among the 3 groups (Group 1: control patients, group 2: AS patients with mild and moderate proteinuria, group 3: AS patients with heavy proteinuria. Magnifications: a-c, e-g and i-k were 400, and the scar bar in 1a is 50μm and applicable to all of the 9 immunofluorescence Figures. *, *p*< 0.5; **, *p*<0.01; ***, *p*< 0.001).

Immunofluorescence revealed that the VEGFA and VEGFR2 expression levels were low in control patients and were slightly increased in AS patients with mild proteinuria. The VEGFA and VEGFR2 expression levels were significantly upregulated in patients with heavy proteinuria and were observed in mass distribution (Table 3, Fig 2a–2h). The expression levels of VEGFA and VEGFR2 were positively correlated with the degree of proteinuria in AS patients, respectively (Table 4). The VEGFA expression levels were positively correlated with the levels of Scr and BUN. However, the VEGFA and VEGFR2 expression levels were not correlated with GFR (Table 4).

**Table 3:** Comparison of the immunofluorescence optical density of VEGFA, VEGFR2, p-VEGFR2 p-nephrin, synaptopodin and nephrin [median (maximum, minimum)] in control and AS patients with different degree of proteinuria.

	VEGFA	VEGFR2	p-VEGFR2	synaptopodin	nephrin	p-nephrin
Group 1	10.6 (6.6, 16)	6.9 (0.0, 22.5)	0.0 (0.0, 8.6)	77.2 (74.5, 84.3)	79.4 (69.9, 84.1)	0.0 (0.0, 23.4)
Group 2	0.0 (0.0, 28.2)	14.2 (0.0, 50.9)	7.9 (0.0, 38.4)	69.0 (56.9, 79.6)	65.2 (52.8, 83.4)	0.0 (0.0, 40.5)
Group 3	55.5 (22.3, 73.8)	40.8 (18.4, 82.1)	56.4 (13.9, 82.4)	40.9 (27.7, 67.2)	32.4 (20.2, 44.5)	62.5 (16.4, 77.8)
Group 1 *vs*. Group 2	*p* = 0.015	*p* = 0.351	*p* = 0.013	*p* = 0.006	*p* = 0.01	*p* = 0.198
Group 1 *vs*. Group 3	*p* < 0.001	*p* < 0.001	*p* < 0.001	*p* < 0.001	*p* < 0.001	*p* < 0.001
Group 2 *vs*. Group 3	*p* < 0.001	*p* = 0.001	*p* < 0.001	*p* < 0.001	*p* < 0.001	*p* < 0.001

Note: Group 1, control patients; group 2, patients with mild and moderate proteinuria; group 3; patients with heavy proteinuria.

**Table 4:** Correlation analyses between the indexes of renal impairments (degrees of proteinuria, Scr, BUN and GFR) and the expression levels of VEGFA, VEGFR2, synaptopodin and nephrin as well as the levels of p-nephrin and p-VEGFR2 in AS patients.

	VEGFA	VEGFR2	p-VEGFR2	synaptopodin	nephrin	p-nephrin
Proteinuria	*r* = 0.814, *p* <0.001	*r* = 0.581, *p* = 0.002	*r* = 0.796, *p* <0.001	*r* = -0.608, *p* = 0.001	*r* = -0.825, *p* <0.001	*r* = 0.737, *p* <0.001
Scr	*r* = 0.600, *p* = 0.002	*r* = 0.321, *p* = 0.117	*r* = 0.627, *p* = 0.001	*r* = -0.660, *p* = < 0.001	*r* = -0.612, *p* = 0.001	*r* = 0.511, *p* = 0.009
BUN	*r* = 0.622, *p* = 0.013	*r* = 0.222, *p* = 0.286	*r* = 0.535, *p* = 0.006	*r* = -0.612, *p* = 0.001	*r* = -0.613, *p* = 0.001	*r* = 0.455, *p* = 0.022
GFR	*r* = -0.264, *p* = 0.202	*r* = -0.076, *p* = 0.717	*r* = -0.352, *p* = 0.085	*r* = 0.325, *p* = 0.112	*r* = 0.185, *p* = 0.377	*r* = -0.040, *p* = 0.849

Note: Scr, serum creatinine; BUN, blood urine nitrogen; GFR, glomerular filtration rate.

The fluorescence of p-VEGFR2 was faint or undetectable in control patients and in some patients with mild proteinuria (5 cases); the fluorescence of p-VEGFR2 was slightly increased in patients with moderate proteinuria and became bright in patients with heavy proteinuria (Table 3, Fig 2i–2l). The level of p-VEGFR2 was positively correlated with the degree of proteinuria in AS patients (Table 4). The levels of p-VEGFR2 were positively correlated with the levels of Scr and BUN, but they showed no relationship with levels of GFR (Table 4).

#### The expression of synaptopodin and nephrin and the phosphorylation of p-nephrin in glomeruli and their association with proteinuria, Scr, BUN and GFR in AS patients

**Fig 3:**
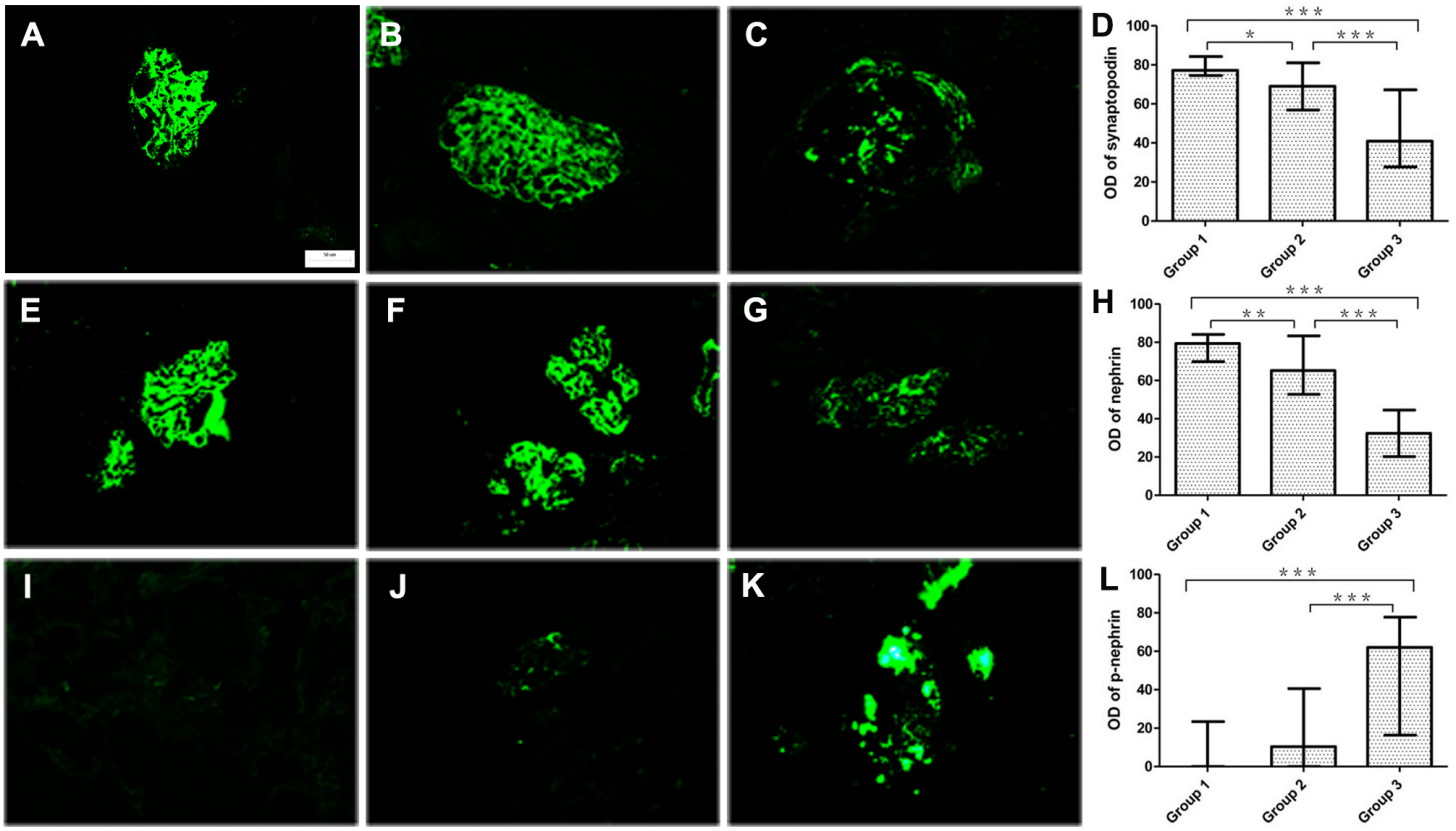
The expression of synaptopodin, nephrin and p-nephrin in glomeruli (immunofluorescence, a, e and i: control patients; b, f and j: AS patients with mild proteinuria; and c, g and k: AS patients with heavy proteinuria). The expression of synaptopodin and nephrin were apparent and regular in both control patients (a and e) and AS patients with mild proteinuria (b and f), but it was prominently down-regulated in AS patients with heavy proteinuria (c and g). The level of p-nephrin was low or undetectable in control patients and in AS patients with mild proteinruia (i and j), and it was significantly up-regulated in AS patients with heavy proteinuria (k). d, h and l were comparison analyses of the immunofluorescence optical density of synaptopodin, nephrin and p-nephrin for the 3 groups (Group 1: control patients, group 2: AS patients with mild and moderate proteinuria, and group 3: AS patients with heavy proteinuria. Magnifications: a-c, e-g and i-k were 400, and the scar bar in 1a is 50μm and applicable to all of the 9 immunofluorescence Figures. *, *p*< 0.5; **, *p*<0.01; ***, *p*< 0.001).

Immunofluorescence revealed that the expression levels of synaptopodin and nephrin in the glomeruli were high and regular and appeared linear or reticular in control patients. The expression levels of synaptopodin and nephrin were slightly decreased in patients with mild and moderate proteinuria, and their expression levels were significantly down-regulated in patients with heavy preoteiuria (Table 3, Fig 3a–3h). The expression levels of synaptopodin and nephrin were negatively correlated with the degree of proteinuria and the levels of Scr and BUN in AS patients, but were not correlated with the levels of GFR (Table 4).

The fluorescence of p-nephrin was faint or undetectable in control patients and in some patients with mild proteinuria (3 cases). The fluorescence of p-nephrin was significantly increased in patients with heavy proteinuria (Table 3, Fig 3i–3l). The level of p-nephrin was positively correlated with the degree of proteinuria and the levels of Scr and BUN in AS patients, but they were not correlated with GFR (Table 4).

#### The association between VEGFA, VEGFR2, p-VEGFR2, synaptopodin, nephrin and p-nephrin (Table 5)

**Table 5:** Correlation analyses between podocyte injuries and the expression level of VEGFA, the activation of the VEGFA-VEGFR2 and the nephrin signaling pathways.

	synaptopodin	nephrin	p-nephrin	VEGFA
VEGFA	*r* = -0.806	*r* = -0.772	*r* = 0.658	—
	*p* < 0.001	*p* < 0.001	*p* < 0.001	—
VEGFR2	*r* = -0.355	*r* = -0.462	*r* = 0.694	*r* = 0.532
	*p* = 0.029	*p* = 0.005	*p* < 0.001	*p* = 0.001
p-VEGFR2	*r* = -0.769	*r* = -0.790	*r* = 0.682	*r* = 0.738
	*p* < 0.001	*p* < 0.001	*p* < 0.001	*p* < 0.001

The VEGFA and VEGFR2 expression levels and the level of p-VEGFR2 were negatively correlated with the expression levels of synaptopodin and nephrin, but they were positively correlated with the level of p-nephrin in glomeruli. The VEGFA expression level was positively correlated with the expression level of VEGFR2 and the level of p-VEGFR2.

## Discussion

AS is a progressive inherited glomerulonephritis [1, 2]. Hematuria is the initial presentation at early stage [13]. Proteinuria emerges with disease progression [3]. AS could be evidenced by the lack of COL IV α3 chain or α5 chain in GBM. In progressed AS patients, electron microscopy revealed distinct structural changes of the GBM; the GBM became thinner, thicker, layered or splitting [14]. In the present study, all patients were diagnosed by their lacking of COL IV α5 and/or α3 chain in GBM, and/or typical GBM changes of AS under EM. Both the levels of Scr and BUN were correlated with the levels of proteinuria in the present study, which indicated that proteinuria was associated with the deterioration of renal function. Although the GFR levels were not correlated with proteinuria which may attribute to the small number of patients.

VEGFA is a key factor for the development and maturation of the GFB and for the maintenance of GFB function [15]. VEGFA in GFB mainly comes from podocytes [16, 17]. Either over-expression or down-regulation of VEGFA would lead to damage of the GFB and the development of proteinruia. The up regulation of VEGFA in the podocytes of mice could cause proteinuria, fusion of the foot precess, effacement of the diaphragm and down-regulation of nephrin [18]. Increased expression of VEGFA in glomeruli was also detected in minimal change of disease, membranous nephropathy, mesangial proliferative glomerulonephritis, lupus nephritis, and diabetic nephropathy [19]. In rats with adriamycin nephropathy or puromycin progressive nephritis, decreased expression of VEGFA in the glomeruli was observed and was associated with the development of proteinuia [5]. Clinically, the use of anti-VEGFA antibodies in patients with neoplastic diseases could cause proteinuria [19].

In the present study, the expression of VEGFA in glomeruli was up-regulated in AS patients, especially in patients with heavy proteinuria. The expression level of VEGFA was positively correlated with the degree of proteinuria and the levels of Scr and BUN in AS patients. Immune electron microscopy revealed that increased accumulation of VEGFA was observed within the three layers of the GFB (podocytes, GBM and glomerular endothelial cells) in AS patients. The accumulation of VEGFA in the GBM was prominent in patients with heavy proteinuria. These results indicated that VEGFA was over-expressed in glomeruli and was associated with the development of proteinuria in AS patients. However, the mechanism remains unclear.

AS is characterized by defect in COL IVα3 or α5in GBM [1, 2, 19]. At the early stage, AS patients manifest with microhematuria and the GBM appears as normal under electron microscopy; in advanced patients, proteinuria develops and the GBM shows distinct structural changes; such as attenuation, thicken, layering or splitting [14]. These changes may affect the function of the GBM as the support of the glomerular capillary as well as the signal crass-talk platform between the glomerular endothelial cells and podocytes [20]. Whether these changes would affect the expression of VEGFA and its distribution in GBM deserves more studies.

VEGFR2 is the main receptor of VEGFA [21], and it plays a role in regulating angiogenesis, mitogenic signaling and permeability-enhancing effects [6]. VEGFR2 is expressed both in the podocytes and endothelial cells of glomeruli, and VEGFA plays its role by autocrine and paracrine pathways after being secreted from podocytes [22, 23]. However, controversy exists regarding whether VEGFR2 is expressed in podocytes in *vivo* [24]. In the present study, we evidenced by immune electron microscopy that VEGFR2 was expressed in podocytes both in control and AS patients. Immunofluorescence showed that the expression of VEGFR2 and the level of p-VEGFR2 were increased in AS patients, especially in patients with heavy proteinuria. These results indicated that the VEGFA-VEGFR2 signaling pathway was activated in AS patients. Moreover, we found that the expression of VEGFR2 in glomeruli was positively correlated with the expression of VEGFA in AS patients. It has been reported that increased expression of VEGFA could induce up-regulation of VEGFR2 in podocytes in *vitro* [24]. In AS patients, whether the up-regulation of VEGFR2 is induced by the up-regulation of VEGFA deserves further study.

VEGFR2 can bind to the intracellular domains of nephrin [7]. Nephrin is the skeleton of the foot process diaphragm of the podocytes as well as a key signal transductor [25]. After binding with VEGFA, VEGFR2 could be phosphorylated, alter the binding status with nephrin and recruit Nck to format VEGFR2-nephrin-Nck complex, which plays a role in regulating the reorganization and polymerization of actin and lead to changes in podocyte modality [7]. Podocyte injury has been proven to be associated with the development of proteinuria in AS. Ding *et al* [11] has reported that the width of the foot process was positively correlated with the degree of proteinuria in AS patients, and the expression levels of synaptopodin and nephrin were negatively correlated with the degree of proteinuria and the levels of Scr and BUN. Previously, we also found that the development of proteinuria was associated with podocyte injury; the degree of foot process fusion was more severe in patients with persistent proteinuria than in patients with mild proteinuria [26]. Synaptopodin is an important structural protein of podocytes and it plays a significant role in maintaining the stability of podocyte skeleton [27]. In the present study, we also found that the expression of synaptopodin and nephrin were down-regulated and negatively correlated with the degree of proteinuria, and the distribution of synaptopodin and nephrin was disturbed in AS patients with heavy proteinuria. Furthermore, we found that the level of p-nephrin was low in the control group and AS patients with mild proteinuria, but it was prominently increased in patients with heavy proteinuria and it was also positively correlated with the degree of proteinuria. These results indicated that the activation of the nephrin signaling pathway is associated with podocyte injury and the development of proteinuria in AS.

It has been reported that over-expression of VEGFA in podocytes could lead to phosphorylation of VEGFR2, down-regulation of nephrin and proteinuria in mice [4, 28]. Phosphorylation of VEGFR2 (Y1214) could recruit Nck, activate the Fyn signaling pathway, and enhance the phosphorylation of nephrin [29]. In the present study, we also found that the expression of VEGFA and the level of p-VEGFR2 were positively correlated with the level of p-nephrin, but they were negatively correlated with the expression of synaptopodin and nephrin, indicating that up-regulated VEGFA expression in glomeruli may activate the VEGFA-VEGFR2 signaling pathway, which would then activate the nephrin signaling pathway and cause podocyte injury and the development of proteinuria in AS patients.

## Conclusion

In conclusion, the expression of VEGFA in glomeruli and its accumulation within GBM were increased as were the levels of p-VEGFR2 and p-nephrin, all of which were positively correlated with the degree of proteinuria in AS patients. The expression levels of synaptopodin and nephrin were down-regulated and negatively correlated with degree of proteinuria. These results indicated that the defect of the GBM in AS may lead to over-expression of VEGFA in the glomeruli and its accumulation in GBM, which could occur through the activation of the VEGFA-VEGFR2 and nephrin signaling pathways, leading to podocyte injury and the development of proteinuria.
